# Invited Discussion "From the SAFE to the SAFEST Liposuction: Combining PAL and RFAL Technology in Body Contouring Procedures"

**DOI:** 10.1007/s00266-023-03792-3

**Published:** 2024-01-12

**Authors:** Fadi Hamadani, Spero J. V. Theodorou

**Affiliations:** 1 Division of Plastic & Reconstructive Surgery, H Clinic Hospital, Ramallah, 9730000 Palestine; 2 Clinical Assistant Professor of Surgery, Aesthetic Plastic Surgery, Zucker School of Medicine Hofstra University, 128 Central Park South, New York, NY 10019 USA

*Level of Evidence V* This journal requires that authors assign a level of evidence to each article. For a full description of these Evidence-Based Medicine ratings, please refer to the Table of Contents or the online Instructions to Authors www.springer.com/00266.

We congratulate Dr. Olivas-Menayo et al. on an original study that has never been described before. In this study, “From the SAFE to the SAFEST liposuction: combining PAL and RFAL technology in body contouring procedures” [1], the authors describe the use of the SAFE liposuction technique followed immediately with the deployment of internal bipolar radiofrequency (RFAL) (Bodytite®, Inmode Ltd., Lake Forrest CA). The stated goal is to achieve tightening of the skin after liposuction. We agree with the authors that internal bipolar radiofrequency has a high affinity for the fibroseptal network (FSN) of the skin which leads to contraction. It also provides the operator with unparalleled temperature and end-point control, elements that have already been established in previous studies [2]. We disagree with their choice to deploy the RFAL after the liposuction, and not before it, for several reasons. Firstly, RFAL relies on an intact FSN to produce the best remodeling, and deploying the RFAL prior to liposuction will ensure that no damage has occurred to the FSN. Although the authors argue that SAFE doesn’t damage the FSN, this is only speculation, and it is best to be cautious and heat first. If one utilizes RFAL after liposuction the flap and FSN architecture are collapsed and hence the target temperature may be artificially elevated due to both the loss of tumescence and fat from the suction. This also lends itself back to a lack of uniform heating with a propensity for end hits and nodules [3]. Secondly, heating the fat prior to liposuction results in much easier extraction, and in our experience negates the need for the “separation” and “equilibration” phases of the SAFE technique, reducing operating times and reducing tissue trauma. It is important to note that if the fat is slated to be injected it is best to perform liposuction followed by heating to avoid potentially damaging the adipocytes with heat.

**Fig. 1:**
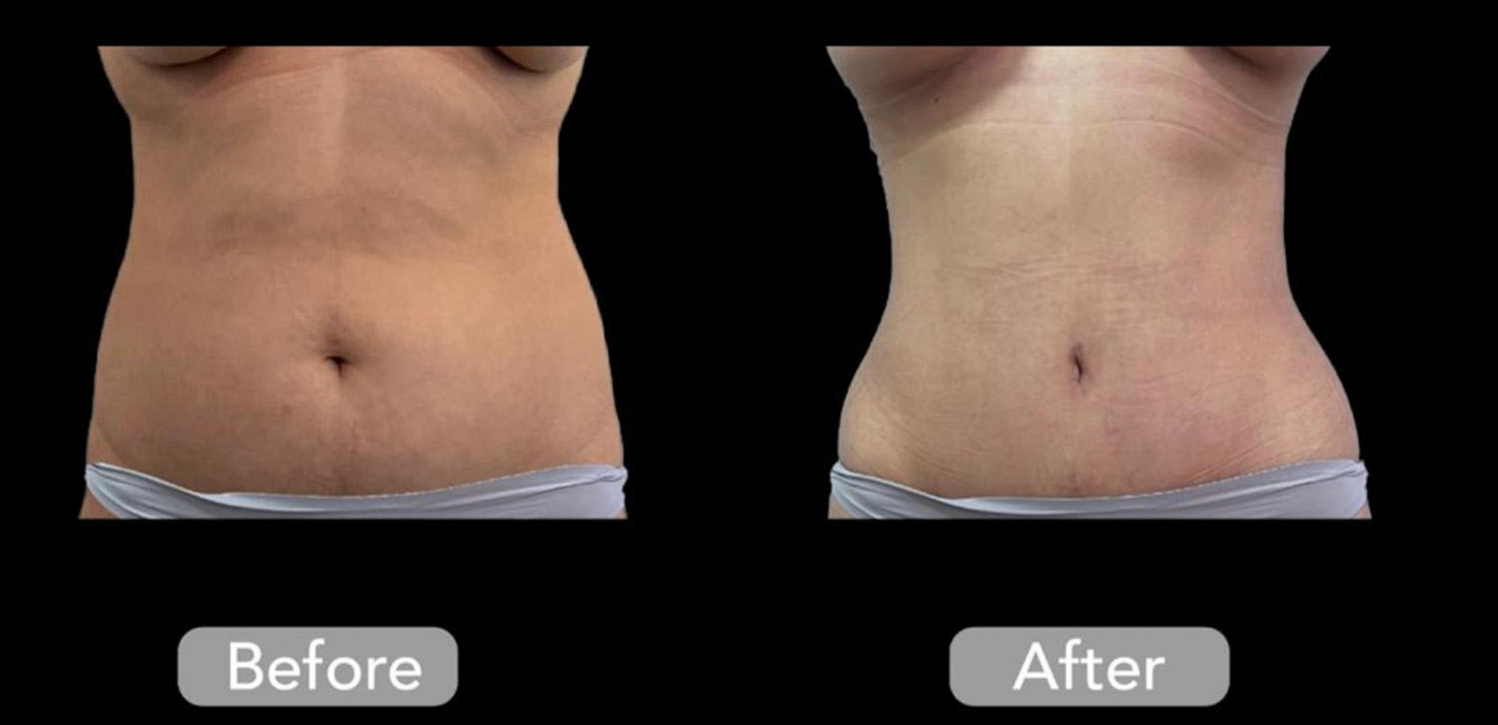
Before and after 3-months of radiofrequency-assisted liposuction and external fractional radiofrequency of the anterior abdomen and flanks. Please note a surgical umbilicoplasty was also performed. Figure courtesy of Fadi Hamadani, MD

The study design was excellent and the authors provided very good follow up to their patients, although the inclusion and exclusion criteria could have been described more clearly. For example, their patient selection was not representative of the full spectrum of cases that RFAL can treat, selecting mostly ideal patients with no preoperative laxity. Our experience has shown that patients with moderate pre-existing laxity are able to benefit significantly from RFAL, often eliminating the need for excisional procedures (Fig. 1). In fact, RFAL is unique among energy-based liposuction in its ability to target the gap population, patients who are in between requiring a definite excisional procedure and those who do not [4]. We would have loved to see the authors apply the technology to patients with more laxity, as the results are impressive.

We have a few comments on the author’s choice of clinical endpoints. To clarify, there are two types of RFAL probes for the body, one with a single external sensor that replaces the sensor in the internal cannula with open slits for the extraction of oils while heating. This handpiece can only measure the external temperature goals and is not available in the US market. The second version, available everywhere including the US, relies on both an internal and external sensor. Total accumulated energy does not necessarily mean that efficient temperature goals were achieved or that the flap was heated uniformly. If the probe is used at the wrong depth or the operator does not move the probe while maintaining excellent coupling between the sensors, it is possible to accumulate a high total energy with no tightening effect at all. It is wasted energy, in a sense. In general, we agree with the authors that an internal temperature endpoint of 65^o^ is a good goal since it is known that collagen contracts at this temperature. However, we disagree with the attempt to quantify energy delivery per body surface area as a dogmatic guideline. Variables such as type of fat, location on body, degree of skin laxity and other factors preclude such stringent recommendations. For example, in RFAL applied to arms, the primary author’s previous study developed a general guideline algorithm that amount of energy deposited is always secondary to temperature endpoint [4]. Once target temperature is achieved the goal of the operator is to make sure that there is uniformity of this target temperature over the whole area treated. This can be achieved by another 1 minute of treatment of the area to ensure that goal [4].

We would like to address the author’s seroma rates which are higher (4.8% and 5.3%) than previously reported with the use of second generation internal RFAL devices (1%) [4]. Slow and gradual heating of tissue with the temperature guidelines of 65^o^ internal and 38^o^ external lead to consistent and uniform results with a low amount of complications [4]. However, high temperatures with a large disruption of the FSN can lead to a high seroma and complication rate [3].

It is important to highlight the role that external, fractional bipolar radiofrequency (Morpheus®, Inmode Ltd., Lake Forrest CA) also has on additional tightening and remodeling of the skin. It would’ve been a strong addition to the current article. This technology, available on the same RFAL platform, is utilized in the same session and provides excellent additional tightening. Dayan et al. have shown the safety of utilizing both internal and external heating in the same session, and we now deploy this procedure in all cases [5].

In summary, this article represents an original concept of combining RFAL with the SAFE liposuction technique and shows that this combination is safe and effective. The authors have utilized the energy based on a sound scientific understanding of skin contraction and have shown excellent results. We believe that heating prior to performing liposuction, although slightly more time-consuming, provides for better contraction and less seroma rates, especially as heating first eliminates the need for equilibration, itself a traumatic and time-consuming step. We feel that the emphasis should be on a uniform volumetric heating of the full thickness of the flap. We congratulate the authors again on a well-designed clinical study which highlights the true power of RFAL in contracting the skin.
